# Online hemodiafiltration vs. high-flux hemodialysis in end-stage renal disease: a meta-analysis

**DOI:** 10.1590/1806-9282.2024D709

**Published:** 2024-09-16

**Authors:** Antonio Silvinato, Idevaldo Floriano, Wanderley Marques Bernardo

**Affiliations:** 1 Brazilian Medical Association, Evidence-Based Medicine – São Paulo (SP), Brazil.; 2 Universidade de São Paulo, Faculty of Medicine – São Paulo (SP), Brazil.

## DESCRIPTION OF THE EVIDENCE COLLECTION METHODS

A systematic review and meta-analysis were conducted following the Preferred Reporting Items for Systematic Reviews and Meta-Analysis statement. Electronic databases including Medline, CENTRAL/Cochrane, LILACS, and ClinicalTrials.gov were searched from inception to May 2024 for randomized controlled trials comparing hemodiafiltration (HDF) and high-flux hemodialysis (HF-HD) in maintenance dialysis patients. The certainty of evidence for each outcome was assessed using the Grading of Recommendation, Assessment, Development, and Evaluation (GRADE) system. It has been registered in PROSPERO [PROSPERO (york.ac.uk)], with the registration number CRD42024563563.

## QUALITY OR CERTAINTY OF EVIDENCE

The certainty of evidence was assessed based on GRADE, graduated in very low, low, moderate, or high.

## GOALS

This study aimed to conduct a meta-analysis to compare online HDF and HF-HD regarding benefits and harms for patients with end-stage renal disease on maintenance dialysis.

## INTRODUCTION

Hemodialysis (HD) and hemodiafiltration (HDF) are different methods of renal replacement therapy (RRT). Hemodialysis can be categorized into low and high flux (HF-HD) based on the pore size of the membrane and the ultrafiltration coefficient of dialyzers. HF-HD is currently considered the standard HD procedure. HDF combines HF-HD with the ultrafiltration of large volumes of plasma water, significantly enhancing the convective transport of substances such as medium and high molecular weight uremic toxins^1-3^. HDF may not be suitable for all patients because it requires a higher blood flow rate to be effective.

In addition to the importance of high-flux hemodiafilters for achieving high volumes of replacement fluids, the dialysis machine plays a substantial role in HDF treatments. The high transmembrane pressure associated with large infusion volumes results in unstable treatment conditions, multiple therapy interruptions, and protein loss through the membrane. The search for the best balance has led to a series of innovations in controlling infusion rates during HDF^4^.

The volume removed during an HDF session is generally much greater than the volume of extracellular fluid itself, making it necessary to infuse almost the entire volume removed throughout the treatment. This large volume of replaced fluid is called the replacement or infusion volume, leaving the patient, at the end of the HDF session, with a body weight close to their dry weight. To obtain this replacement solution volume, with appropriate electrolyte composition, sterile, pyrogen-free, and low-cost, online HDF (OL-HDF) was developed^5^.

In OL-HDF, part of the dialysis solution undergoes double filtration, producing the replacement solution, which will be infused into the patient. The remaining solution, which has not been filtered in this second step, is used as ultrapure dialysis solution for solute removal by diffusion. The technical and clinical aspects of OL-HDF are fundamental to the success of the treatment, including the proper choice of the dialyzer, the configuration of the HDF machine, and the determination of the convective dose. These elements play a crucial role in achieving high replacement volumes during dialysis sessions, contributing to the effectiveness of the treatment^6,7^. The replacement fluid is generally administered in post-dilution mode.

The potential benefits of OL-HDF therapy compared to conventional HD are based on the improved efficiency of solute removal using higher convective doses combined with diffusion^8^. Clinical trial analyses have identified that a high volume of convection is associated with a reduction in mortality, compared to conventional HD^9^ (HF-HD – currently considered the standard procedure for HD^10^). This study aims to gather current data from randomized clinical trials (RCTs) through a meta-analysis comparing HF-HD and OL-HDF in terms of mortality in patients with end-stage renal disease (ESRD) and maintenance dialysis.

## OBJECTIVE

To assess the benefits and harms of OL-HDF in patients with ESRD and maintenance dialysis compared to HF-HD through a systematic review and meta-analysis of RCTs.

## METHODOLOGY

This systematic review followed the Preferred Reporting Items for Systematic Reviews and Meta-analyses (PRISMA)^11^ guidelines and is supported by scientific information obtained through a systematic literature review of published studies. It has been registered in PROSPERO [PROSPERO (york.ac.uk)], with the registration number CRD42024563563.

### Eligibility criteria

The eligibility criteria specify the specific elements to address the clinical question of this evaluation (objective).

### Inclusion criteria for studies

- Patients: those with ESRD on maintenance dialysis.
- Intervention: OL-HDF.
- Comparison: HF-HD (current HD standard).
- Outcomes: clinically relevant efficacy and safety outcomes.
- Study design: parallel design randomized controlled trials.
- Language: no restriction.
- Consulted period: no restriction.
- Full text available.

Excluded studies: Systematic reviews with or without meta-analysis; narrative reviews; observational studies; and/or case series or studies lacking extractable data (absolute numbers and/or means).

Search for evidence: The search for evidence will be conducted in the Medline virtual scientific database using the search strategy: (Kidney Failure, Chronic OR Chronic Renal Insufficiencies OR Chronic Renal Insufficiency OR Kidney Insufficiency Chronic OR Chronic Kidney Disease OR Chronic Renal Disease) AND (Hemodiafiltration OR on-line hemodiafiltration OR online hemodiafiltration OR OL-HDF) AND Random*; CENTRAL/Cochrane: (Kidney Failure, Chronic OR Chronic Renal Insufficiencies OR Chronic Renal Insufficiency OR Kidney Insufficiency Chronic OR Chronic Kidney Disease OR Chronic Renal Disease) AND (Hemodiafiltration); LILACS: hemodiafiltration AND [db:("LILACS") AND type_of_study:("clinical_trials")] and ClinicalTrials.gov: Hemodiafiltration Study Typ=Interventional (Clinical Trial). Additional manual searches were performed in the reference lists of included studies and other relevant sources. The search in these databases was conducted until May 2024.

### Study selection process and data extraction

The evidence retrieved from the consulted databases is initially selected based on the title and abstract to meet eligibility criteria. The studies meeting these criteria in the initial selection have their full texts accessed to confirm eligibility. The retrieval process, as well as the evaluation of titles and abstracts obtained, was conducted independently and blinded by two researchers skilled in systematic reviews (AS and IF), following the inclusion and exclusion criteria. Subsequently, the selected articles were critically evaluated for inclusion in the review. In cases of disagreement between the researchers regarding study selection, a third reviewer (WMB) was consulted.

From the eligible studies, the following data will be extracted: author's name and year of publication, study population, intervention and comparison methods, and follow-up time. Regarding the extracted data for relevant outcomes, depending on the type of outcome, these may include numbers of events or means and/or medians, with their respective standard deviations or 95% confidence intervals (CIs).

### Risk of bias and quality of evidence

Two independent reviewers assessed the risk of bias in the included studies using the Cochrane Risk of Bias Tool for randomized trials (RoB 2)^12^, supplemented with additional key elements, and expressed as high, moderate, or low. Each domain was classified as low bias, unclear bias, or high bias.

Publication bias was assessed using the funnel plot inspection and Egger^13^ test. A p-value<0,05 was considered as evidence of statistically significant publication bias. All statistical tests were two-tailed.

The Grading of Recommendation, Assessment, Development, and Evaluation (GRADE)^14^ criteria were used to assess the certainty of the pooled evidence, classifying the quality of evidence into four levels: high, moderate, low, and very low. Two reviewers assessed the risk of bias, inconsistency, indirect evidence, imprecision, and publication bias for all reported outcomes. The quality of evidence was evaluated using the "Guideline Development Tool" (GRADEpro GDT)^15^ and presented in GRADE evidence profiles and summary of finding tables using standardized terminology.

### Method of analysis and synthesis of results

The data will be analyzed according to the intention-to-treat (ITT) principle, and the most recent available follow-up data were included in each trial.

The results for categorical outcomes will be expressed using the risk difference (RD) between the intervention and control groups, employing the Mantel–Haenszel method. If the RD between groups is statistically significant (95% confidence), it will be reported along with the 95% CI and the Number Needed to Treat (NNT) or Number Needed to Harm (NNH).

If there are multiple studies included with common outcomes, these will be aggregated through meta-analysis using Review Manager 5.4 (The Nordic Cochrane Centre, The Cochrane Collaboration)^16^. The overall RD with 95%CIs will be the final measure used to support evidence synthesis, addressing the clinical question (objective). For studies reporting data as medians and interquartile range, the statistical formula proposed by Hozo et al.^17^ was used to estimate means and standard deviations.

Additionally, statistical analysis will be conducted using the "meta" package in the R programming language (version 4.3.2; R Core Team 2023, Vienna, Austria)^18^, with the "metainc" function employed to analyze data from studies reporting hospitalization rates. To explain the magnitude effect, we will report an incidence rate ratio (IRR) with a 95%CI. A significance level of p<0.05 was considered statistically significant.

The estimation of combined effect sizes will be conducted using a fixed-effect or random-effect model depending on the assessment of heterogeneity in the results. Statistical heterogeneity (inconsistency) was evaluated using the I^2^ metric, which measures the percentage of variation across studies, due to heterogeneity rather than random chance^19^. Heterogeneity values above 50% were considered substantial.

Sensitivity analysis was conducted to assess the reliability of the study finding. We used a funnel plot for asymmetry analysis, which was evaluated after excluding outliers.

### Evidence synthesis and conclusion

The evidence synthesis will present the results directly from the analyses, considering benefit, harm, and lack of difference between OL-HDF compared in parallel with HF-HD. Conclusions will consider evidence of at least moderate quality, the presence of effect, whether it is beneficial or harmful, and the balance between favorable benefits and harms in patients with ESRD undergoing maintenance dialysis.

## RESULTS

In the search for evidence on the use of OL-HDF, the following numbers of studies were retrieved from the databases: 255 from MEDLINE, 314 from CENTRAL, 2 from LILACS, and 28 from ClinicalTrials.gov. No studies were retrieved from manual and/or gray literature searches.

After removing duplicates and excluding studies based on title and/or abstract screening, 12 studies remained that met the pre-established eligibility criteria (methodology). These 12 studies were selected for full-text access. Following the review of the full texts, six randomized controlled trials conducted in parallel with HF-HD^20-25^ were included to support the conclusions of this assessment.

**Figure 1:**
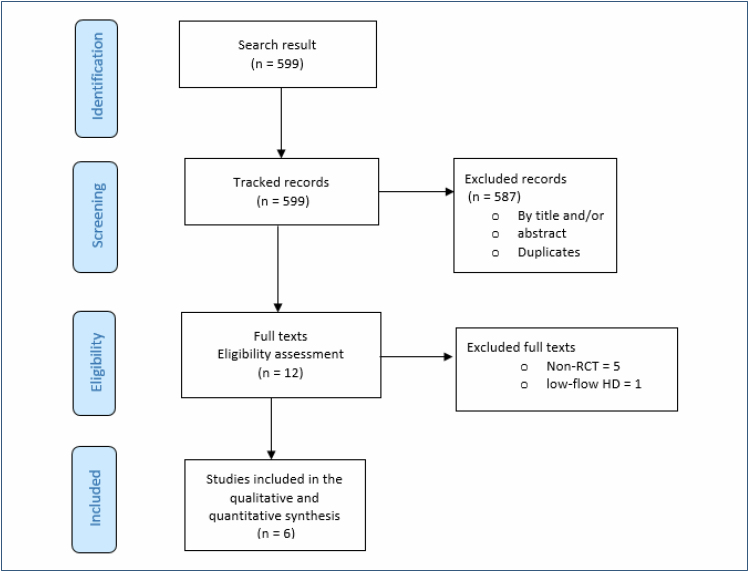
Flow diagram representing the process of study selection.

Source: Moher D, Liberati A, Tetzlaff J, Altman DG, The PRISMA Group (2009). Preferred Reporting Items for Systematic Reviews and Meta-Analyses: The PRISMA Statement. PLoS Med 6(7): e1000097. doi:10.1371/journal. pmed1000097

The reasons for excluding the other six studies were: not being RCTs; comparing OL-HDF with low-flow HD (not the current standard of HD^10^) (Figure 1). The references for the excluded studies, along with the reasons for their exclusions, as well as the references for the ongoing studies, are in Appendices 3 and 4. The flow diagram illustrating the sequence from retrieval to selection of evidence to support this assessment is in Figure 1.

The key baseline characteristics and details of each included trial are reported in Appendice 1. These trials included 3,629 participants (1,821 randomized to OL-HDF and 1,808 to the HF-HD group).

### Bias risk in the studies

Regarding the bias risk of the six included^20-25^: One had nuclear randomization (use of coin) and allocation concealment^25^; none were double-blinded; one did not blind the outcome assessor^25^, and in three, blinding of the outcome assessor was uncertain as it was unclear to whom blinding referred^22-24^; four studies had losses greater than 20%^21-24^; one did not show similar baseline characteristics between the two treatments^21^; one did not conduct ITT analysis;^23^ and one did not perform the sample size calculation^25^. One clinical trial was considered to have a low overall risk of bias^20^, and the other five had high risk^21-25^. The assessment of bias risk for each individual study, conducted using the RoB2^12^ tool supplemented with other key elements, is reported in Table 1.

**Table 1:** Risk of bias in studies.

First author/year (Ref. No.)	Randomization	Blind allocation	Double-blind	Outcome researcher blind	Losses	Prognostic characteristics	Appropriate outcomes	Intention-to-treat analysis	Sample size calculation	Early interruption	Overall risk of bias
Blankestijn et al. 2023 (^20^)											Low
Kang et al.2021 (^21^)											High
Morena et al. 2017 (^22^)											High
Ok et al. 2013 (^23^)											High
Maduell et al. 2013 (^24^)											High
Schiffl 2007 (^25^)											High

Legend	Low Risk		Not Informed		High Risk						

### Outcomes

The evidence levels for each outcome, according to the GRADE system, are provided in Appendice 2.

#### All-cause mortality

**Figure 2:**
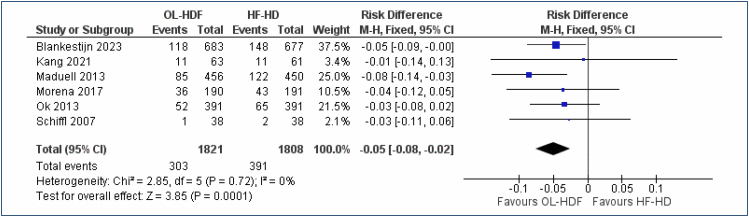
Forest plot of the comparison: 1 online hemodiafiltration versus high-flux hemodialysis, outcome: 1.1 All-cause mortality at 2–3 years.

Six studies^20-25^, with a total of 3,629 participants, allowed for the evaluation of the outcome "all-cause mortality," comparing OL-HDF versus high-flux HD, with follow-up periods of 2–3 years. This analysis showed a 5% reduction in the risk of death (RD=5% [95%CI, 2–8%]; I^2^=0%; p=0.0001) with the use of OL-HDF compared to high-flux HD, requiring treatment of 20 patients (NNT=20) to prevent one death, with a possible range of 13–50 patients (95%CI, 13–50) (Figure 2). The Egger test (funnel plot) did not identify any outlier studies. The quality of evidence is low.

#### Cardiovascular mortality

**Figure 3:**
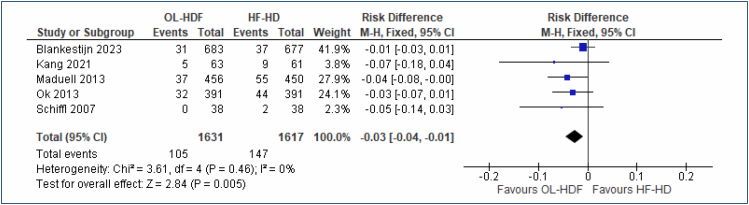
Forest plot of the comparison: 1 online hemodiafiltration versus high-flux hemodialysis, outcome: 1.2 Cardiovascular mortality at 2–4 years.

Five studies^20,21,23-25^, including a total of 3,248 participants, evaluated the outcome "cardiovascular mortality," comparing OL-HDF versus high-flux HD with follow-up periods of 2–4 years. In this comparison, OL-HDF reduced the risk of cardiovascular death by 3% (RD=3% [95%CI, 1–4%]; I^2^=0%; p=0.005; NNT=33 [95%CI, 25–100]) (Figure 3). The Egger test did not identify any outlier studies. The quality of evidence is very low.

#### Infection mortality

**Figure 4:**
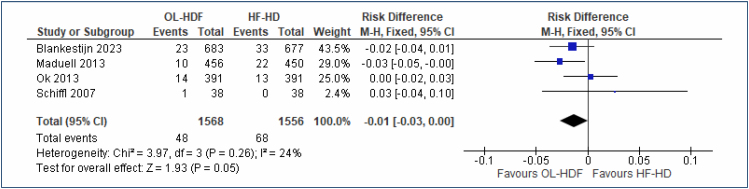
Forest plot of the comparison: 1 online hemodiafiltration versus high-flux hemodialysis, outcome: 1.3 Infection mortality at 2–3 years.

Four studies^20,23-25^, with a total of 3,124 participants, evaluated the outcome "infection mortality," comparing OL-HDF versus high-flux HD, and showed no difference between the two procedures over a follow-up period of 2–3 years (RD=-0.01 [95%CI, -0.03 to 0.00]; I^2^=24%, p=0.05) (Figure 4). The funnel plot did not identify any outlier studies. The quality of evidence is low.

#### Fatal and nonffatal cardiovascular events

**Figure 5:**
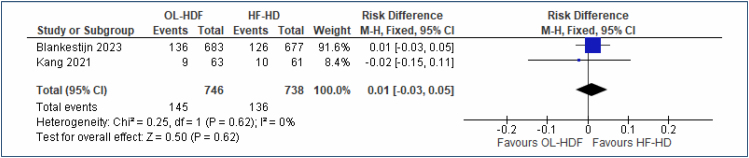
Forest plot of the comparison: 1 online hemodiafiltration versus high-flux hemodialysis, outcome: 1.4 fatal and nonfatal cardiovascular events at 3–4 years.

This is a composite outcome of cardiovascular death or any of the following events requiring or occurring during hospitalization: acute myocardial infarction, stroke, percutaneous coronary or cerebrovascular revascularization, or surgical coronary or cerebral revascularization. Two studies^20,21^ including a total of 1,484 participants assessed this outcome, and meta-analysis showed no difference between OL-HDF and HF-HD (RD=1% [95%CI, -0.03 to 0.05]; I^2^=0%; p=0.62) (Figure 5). The funnel plot did not identify any outlier studies. The quality of evidence is moderate.

#### Hospitalizations

**Figure 6:**
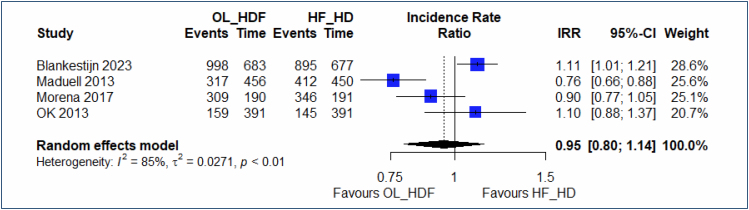
Forest plot of the comparison: 1 online hemodiafiltration versus high-flux hemodialysis, outcome: 1.5 hospitalizations at 2−3 years.

Four studies^20,22-24^ allowed for the evaluation of the outcome "hospitalization," comparing OL-HDF and high-flux HD over a follow-up period of 2–3 years. The results indicate that, despite high heterogeneity among the studies (I^2^=85.5% [64.2%; 94.1%]), the combined estimate of hospitalization rates between the two groups is not significantly different. The relative incidence rate shows no difference between the groups (IRR=0.95 [0.79; 1.13]; p=0.58) (Figure 6). The funnel plot did not identify any outlier studies. The quality of evidence is low.

## EVIDENCE SYNTHESIS

In patients with ESRD undergoing maintenance dialysis, OL-HDF compared to HF-HD:

- Reduces the risk of all-cause mortality by 5% (95%CI, 2–8%), with an NNT of 20 to prevent one death, ranging from 13 to 50 patients (95%CI, 13–50), over a follow-up period of 2–3 years. The quality of evidence is low.
- Reduces the risk of cardiovascular mortality by 3% (95%CI, 1–4%), with an NNT of 33 (95%CI, 25–100), over a follow-up period of 2–4 years. The quality of evidence is very low.
- Shows no difference in the risk of mortality due to infection (RD=-0.01 [95%CI, -0.03 to 0.00]; p=0.05), over a follow-up period of 2–3 years. The quality of evidence is low.
- There is no difference in the risk of fatal and nonfatal cardiovascular events (RD=1% [95%CI, -0.03 to 0.05]; p=0.62), over a follow-up period of 3–4 years. The quality of evidence is moderate.
- There is no difference in the rate of hospitalizations (IRR=0.95 [95%CI, 0.79–1.13]; p=0.58), over a follow-up period of 2–3 years. The quality of evidence is low.

The evidence levels for each outcome according to the GRADE system are provided in Appendice 2.

## DISCUSSION

The results of the CONTRAST (Convective Transport Study)^26^ showed that for the general population of patients with chronic HD, switching from low-flux HD to OL-HDF may not significantly impact overall mortality or cardiovascular event rates. However, high-volume HDF may offer survival advantages for certain patients, suggesting the need for individualized treatment approaches.

This review contributes to the ongoing debate on the ideal dialysis modality for ESRD patients. It highlights the importance of personalized medicine and the potential benefits of high-volume hemodiafiltration compared to conventional HD^9^ (HF-HD – considered the current standard procedure for HD^10^).

Following a systematic literature search and screening, six RCTs^20-25^ were finally included in the meta-analysis, evaluating the benefits and harms of OL-HDF in patients with ESRD undergoing maintenance dialysis compared to HF-HD. These trials involved a total of 3,629 participants, with 1,821 randomized to OL-HDF and 1,808 to the HF-HD group. Compared to HF-HD, OL-HDF reduced the risk of all-cause mortality by 5% (95%CI, 2–8%). It would be necessary to treat 20 patients (NNT=20) to prevent one death, with a range of 13–50 patients (95%CI, 13–50), over a follow-up of 2–3 years with low quality of evidence. In the same comparison, OL-HDF reduced the risk of cardiovascular mortality by 3% (95%CI, 1–4%), with an NNT of 33 (95%CI, 25–100), over a follow-up of 2–4 years, with very low quality of evidence.

There was no difference between these two procedures for the outcomes: mortality due to infection (low quality of evidence), fatal and nonfatal cardiovascular events (moderate quality of evidence), and hospitalizations (low quality of evidence), during follow-ups of 2–3, 3–4, and 2–3 years, respectively. In general, our primary outcomes are characterized by either zero or highly acceptable heterogeneity.

A strong point of the study was the exclusive inclusion of RCTs. Additionally, we focused on OL-HDF and excluded other convective therapies (HDF or offline HF) to reduce variability in effectiveness among convective modalities. Furthermore, treatment parameters used with OL-HDF were relatively consistent across all included studies. For instance, only one multicenter study^21^, conducted over 1 year within a 4-year period, used pre-dilution HDF. Therefore, the majority of HDF sessions were performed in the post-dilution mode. The other five studies^20,22-25^ also exclusively employed the post-dilution mode.

In five of these studies, the average convection volume was over 17 L per session, ranging from 17.2 to 24.5 L per session^20-24^. One study did not report the convection volume^25^; however, excluding this study from the analyses did not change the evaluated outcomes. In all studies, comparison with OL-HDF included only patients on HF-HD.

There are also some limitations in this review. The characteristics of enrolled patients varied among the analyzed studies. For example, Morena et al.^22^ focused solely on elderly patients over 65 years old, and such differences may have influenced the outcomes of this study. Additionally, the follow-up times of the studies ranged from 2 to 4 years, and this difference in the follow-up period may have also affected the results of our meta-analysis. The high risk of bias in the studies can be considered another limiting factor (see Table 1).

Finally, the number of studies included in this meta-analysis is limited, necessitating additional studies with adequate statistical power to draw more solid conclusions. The conclusion of RCT H4RT (High-volume HDF versus High-flux HD Registry Trial; see References – ongoing studies), expected in 2025, may definitively address the remaining questions.

## CONCLUSION

This meta-analysis indicates that while OL-HDF may offer modest survival benefits compared to HF-HD for patients with ESRD on maintenance dialysis, the choice of dialysis modality should be personalized, considering individual patient characteristics, specific case details, and resource availability. The quality of the evidence ranges from low to very low, suggesting that more high-quality studies are needed to confirm these findings. The anticipated conclusion of the H4RT clinical trial may provide more definitive evidence to guide future clinical practices.

## APPENDICES

### Appendice 1 Characteristics of included studies.

**Table:**

Author	RRT	HDF Convective volume	Population	OL-HDF group (N)	HD group (N)	Outcomes	Follow-up (months)
**Blankestijn et al. 2023** **(CONVINCE Study)** ^20^	OL-HDF versus HF-HD	The target volume of ≥ 23 ± 1 L/session for high-dose convection was achieved in 92% of the HD sessions performed.	Adult patients with stage V renal failure on chronic intermittent HD for at least 3 months.	683	677	All-cause mortality Cardiovascular mortality Composite outcome of fatal or nonfatal cardiovascular events Hospitalizations Death due to infection	30
**Kang et al. 2021** **(FINESSE study)** ^21^	OL-HDF versus HF-HD	Average of 24.5 ± 3.1 L/session	Adult patients with ESKD requiring maintenance HD (incident or prevalent), suitable for any of the interventions	63	61	All-cause mortality Cardiovascular mortality Composite outcome of fatal or nonfatal cardiovascular events	48
**Morena et al. 2017** **(FRENCHIE Study)** ^22^	OL-HDF versus HF-HD	Average of 22.53 ± 6.76 L/session	Elderly patients without significant diuresis (<100 mL/24 h) and/or residual renal function (<2 mL/min/1.73 m2), on HF-HD for ≥3 months and considered stabilized, receiving thrice-weekly HD sessions with hemoglobin levels between 9 and 13 g/dL.	190	191	All-cause mortality Hospitalizations	24
**Ok et al. 2013** **(TURKISH HDF Study)** ^23^	OL-HDF versus HF-HD	Average of 17.2 ± 1.3 L (13.5–20.0) L/session; 96,7% of patients were treated with >15 L/session	Adult patients on maintenance HD with thrice-weekly bicarbonate dialysis for a total of 12 h per week	391	391	All-cause mortality Cardiovascular mortality Hospitalizations Death due to infection	24
**Maduell et al. 2013** **(ESHOL study)** ^24^	OL-HDF versus 92% HF-HD, 8% low-flux	Average of 23.7±0.59 L/session	Adult patients with ESKD receiving standard HD three times per week for 3 months	456	450	All-cause mortality Cardiovascular mortality Hospitalizations Death due to infection	36
**Schiffl 2007** ^25^	OL-HDF versus HF-HD	Not informed	Stable clinically adults (aged 32–78 years) with ESKD who have been on conventional HD three times per week for at least 6 months and have a permanent vascular access capable of allowing a blood flow rate of at least 250 mL/min.	38	38	All-cause mortality Cardiovascular mortality Hospitalizations Death due to infection	24

OL-HDF: online hemodiafiltration; HD: hemodialysis; HF: high-flux; HDF: hemodiafiltration; RRT: renal replacement therapy; ESKD: end-stage kidney disease.

### Appendice 2 Levels of evidence – GRADE system.

**Summary of findings:**

OL-HDF compared to HF-HD – benefit/harm

Patient or population: with end-stage kidney disease on maintenance dialysis

Context: Efficacy and safety

Intervention: OL-HDF

Comparison: HF-HD

**Table:**

Outcomes, follow-up (Number of RCTs)	Potential absolute effects (95% CI)		Absolute risk reduction (95% CI)	Certainty of the evidence (GRADE)
	Risk with HF-HD	Risk with OL-HDF		
All-cause mortality, 2–3 years (6)	391/1808 (21.6%)	303/1821 (16.6%)	50 more per 1000 (from 20 more to 80 more)	⨁⨁◯◯ Low^a^
Cardiovascular mortality, 2–4 years (5)	147/1617 (9.1%)	105/1631 (6.4%)	30 more per 1000 (from 10 more to 40 more)	⨁◯◯◯ Very Low^b^,^c^
Infection-related mortality, 2–3 years (4)	68/1556 (4.4%)	48/1568 (3.1%)	10 more per 1000 (from 0 fewer to 30 more)	⨁⨁◯◯ Low^d^
Fatal and nonfatal cardiovascular events, 3–4 years (2)	136/738 (18.4%)	145/746 (19.4%)	10 fewer per 1000 (from 50 fewer to 30 more)	⨁⨁⨁◯ Moderate^e^

CI: Confidence interval.

a Uncertain evaluator blinding in three studies and absent in one study; loss to follow-up exceeding 20% in four studies; absence of ITT analysis in one study; absence of sample size calculation in another study.

b Uncertain evaluator blinding in two studies and absent in one study; loss to follow-up exceeding 20% in three studies; absence of ITT analysis in 1 study; absence of sample size calculation in another study.

c NNH with a very wide 95% CI.

d Uncertain evaluator blinding in two studies and absent in one study; loss to follow-up exceeding 20% in two studies; absence of ITT analysis in one study; absence of sample size calculation in another study.

e Loss to follow-up exceeding 20% and differing prognostic characteristics that may influence this outcome in one study.

**Table:**

Outcomes, follow-up (Number of RCTs)	Relative incidence rate* (95% CI)	Certainty of the evidence (GRADE)
Hospitalizations, 2−3 years (4)	IRR=0.95, (0.79; 1.13)	⨁⨁◯◯ Low^a^,^b^

* IRR: incidence rate ratio; CI: confidence interval.

a Uncertain evaluator blinding in three studies; loss to follow-up exceeding 20% in three studies; absence of ITT analysis in one study.

b High heterogeneity (I^2^=85%).

**Table:**

**GRADE Working Group grades of evidence** High certainty: We are very confident that the true effect lies close to that of the estimate of the effect. Moderate certainty: We are moderately confident in the effect estimate: The true effect is likely to be close to the estimate of the effect, but there is a possibility that it is substantially different. Low certainty: Our confidence in the effect estimate is limited: The true effect may be substantially different from the estimate of the effect. Very low certainty: We have very little confidence in the effect estimate: The true effect is likely to be substantially different from the estimate of effect.

### Appendice 3 Excluded studies – reasons.

**Table:**

Kikuchi K, Hamano T, Wada A, Nakai S, Masakane I. Predilution online hemodiafiltration is associated with improved survival compared with hemodialysis. Kidney Int. 2019;95:929-38. https://doi.org/10.1016/j.kint.2018.10.036. **Non-randomized**
Locatelli F, Karaboyas A, Pisoni RL, Robinson BM, Fort J, Vanholder R, et al. Mortality risk in patients on hemodiafiltration versus hemodialysis: a ‘real-world’ comparison from the DOPPS. Nephrol Dial Transplant. 2018;33:683-9. https://doi.org/10.1093/ndt/gfx277. **Non-randomized**
Mercadal L, Franck JE, Metzger M, Urena Torres P, Cornelissen F, Edet S, et al. Hemodiafiltration versus hemodialysis and survival in patients with ESRD: the French renal epidemiology and information network (REIN) registry. Am J Kidney Dis. 2016;68:247-55. https://doi.org/10.1053/j.ajkd.2015.11.016. **Non-randomized**
Mesaros-Devcić I, Tomljanović I, Mikolasević I, Dvornik S, Vujicić B, Pavletić-Persić M, et al. Survival of patients treated with online hemodiafiltration compared to conventional hemodialysis. Coll Antropol. 2013;37:827-32. **Non-randomized**
Grooteman MP, Dorpel MA, Bots ML, Penne EL, Weerd NC, Mazairac AH, et al. Effect of online hemodiafiltration on all-cause mortality and cardiovascular outcomes. J Am Soc Nephrol. 2012;23:1087-96. https://doi.org/10.1681/ASN.2011121140. **Low-flux hemodialysis**
Vilar E, Fry AC, Wellsted D, Tattersall JE, Greenwood RN, Farrington K. Long-term outcomes in online hemodiafiltration and high-flux hemodialysis: a comparative analysis. Clin J Am Soc Nephrol. 2009;4:1944-53. https://doi.org/10.2215/CJN.05560809. **Non-randomized**

### Appendice 4 Ongoing studies.

**Table:**

Caskey FJ, Procter S, MacNeill SJ, Wade J, Taylor J, Rooshenas L, et al. The high-volume haemodiafiltration vs high-flux haemodialysis registry trial (H4RT): a multi-centre, unblinded, randomised, parallel-group, superiority study to compare the effectiveness and cost-effectiveness of high-volume haemodiafiltration and high-flux haemodialysis in people with kidney failure on maintenance dialysis using linkage to routine healthcare databases for outcomes. Trials. 2022;23:532. https://doi.org/10.1186/s13063-022-06357-y
